# Supplementary material for: Ménière’s Disease: Insights from an Italian Nationwide Survey
Source: Audiol Res. 2023 Feb 28;13(2):160–8. doi: 10.3390/audiolres13020016 (PMC10037628; doi:10.3390/audiolres13020016)
Supplement: Supplementary file 1 [file audiolres-13-00016-s001.zip › audiolres-2215453-supplementary.pdf]

Supplementary Materials: Ménière's Disease: Insights from an Italian Nationwide Survey

Fulvio Mammarella [1], Antonella Loperfido [1], Elizabeth G. Keeling [2], Gianluca Bellocchi [1] and Luca Marsili [3,*]

[1] Otolaryngology Unit, San Camillo Forlanini Hospital,00152 Rome, Italy
[2] School of Life Sciences, Arizona State University, 85281 Tempe, AZ, USA;
[3] Gardner Family Center for Parkinson's Disease and Movement Disorders, Department of Neurology, University of Cincinnati, 45219 Cincinnati, OH, USA
* Correspondence: luca.marsili@uc.edu; Tel./Fax: +1-(513)-558-7643

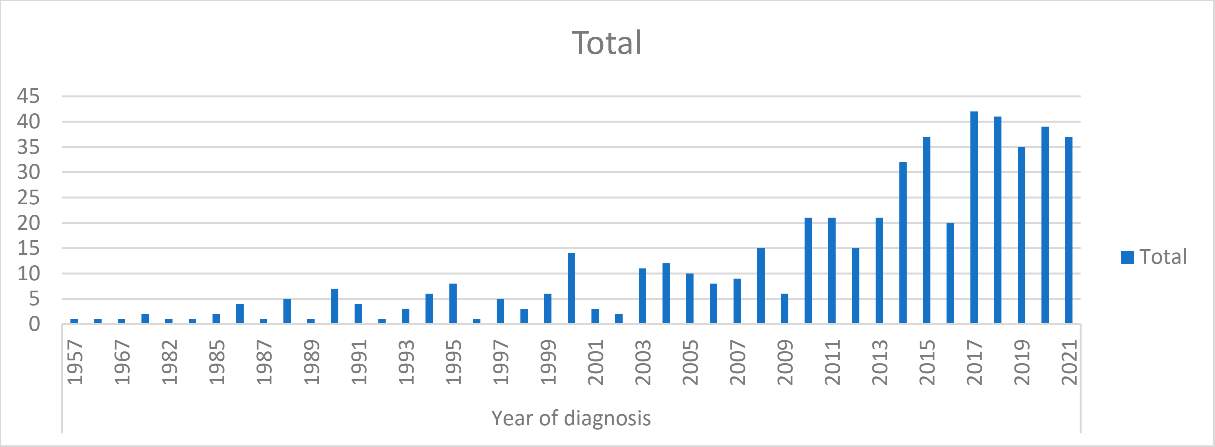

Figure S1. Year of diagnosis.

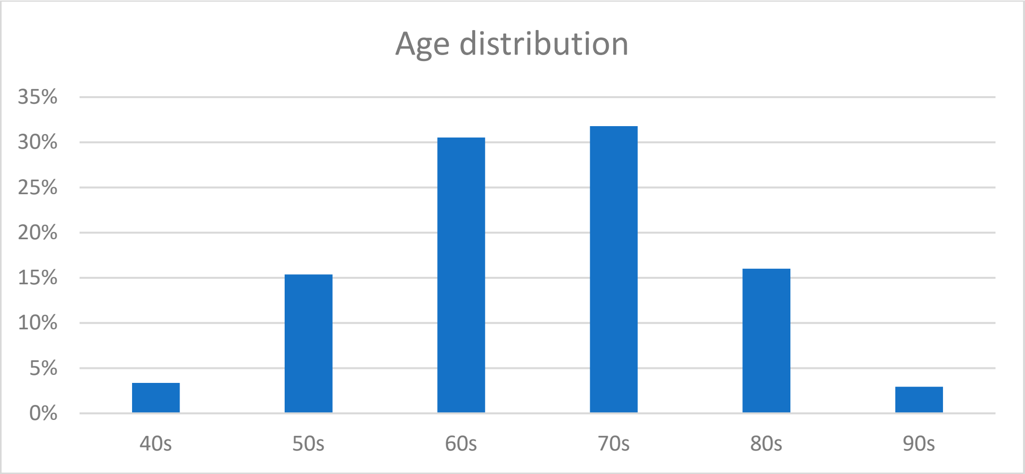

Figure S2. Age distribution.

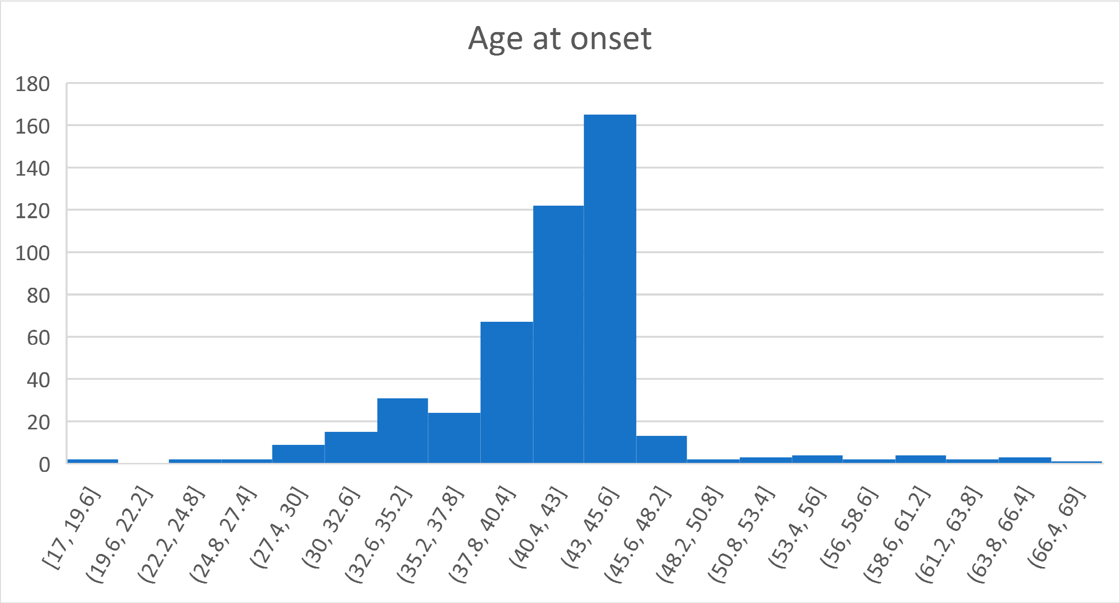

Figure S3. Age at onset.
